# Association between excessive daytime sleepiness, REM phenotype and severity of obstructive sleep apnea

**DOI:** 10.1038/s41598-019-56478-9

**Published:** 2020-01-08

**Authors:** Agata Gabryelska, Piotr Białasiewicz

**Affiliations:** Department of Sleep Medicine and Metabolic Disorders, Medical University of Lodz, Mazowiecka 6/8, 92-215 Lodz, Poland

**Keywords:** Respiratory tract diseases, Outcomes research

## Abstract

The aim of the study was to compare REM-dependent and REM-independent, obstructive sleep apnea syndrome (OSA) patients in relation to their daily sleepiness assessed by Epworth sleepiness scale (ESS). The study included 1863 consecutive patients, who were referred to a sleep centre with a presumed diagnosis of OSA. Following polysomnography, 292 patients fulfilled criteria for either REM-dependent OSA (REM-OSA, n = 102) or REM-independent OSA (nREM-OSA, n = 190). Both study groups were matched regarding sex and age. REM-OSA group had two times lower median apnoea-hypopnea index (AHI) compared to nREM-OSA (p < 0.001), yet day-time sleepiness measured by ESS was similar: median score 9.0 (6.0–11.0) and 8.0 (4.8–11.0), p = 0.109, respectively. Subsequent post-hoc ANCOVA analysis, with covariates (BMI, percent of total sleep time spent in REM stage, percent of total sleep time spent in the supine position), has shown statistically significant difference between study groups regarding AHI (p < 0.001) and no difference regarding ESS score (p = 0.063). Despite two times lower AHI, patients with REM-OSA present with similar day-time sleepiness as those with REM independent OSA. Daily sleepiness may be stronger associated with apneas/hypopneas occurring in REM than nREM sleep.

## Introduction

Obstructive sleep apnea syndrome (OSA) is characterized by recurrent episodes of apneas or hypopneas during sleep leading to intermittent hypoxemia and arousals. The severity of OSA is by convention assessed by apnea–hypopnea index (AHI)^1^. A few distinct phenotypes of OSA have been recognized with the main division lying between patients with sleep disordered breathing (SDB) independent of body position and sleep stage (usually obese with severe disease) and those with SDB dependent on supine position and/or REM sleep (usually non-obese, with mild to moderate disease)^2,3^.

Excessive daily sleepiness (EDS) is the one of leading complaints among patients with OSA and may be assessed by the Epworth sleepiness scale (ESS). It has been shown that EDS leads to impaired concentration, mood lability as well as other neurocognitive difficulties^4^. The relation between ESS score and the severity of OSA is still unclear as literature provides contradictory information with some researchers arguing in favour^5–7^ and some against^8,9^. It is conceivable that EDS may depend not solely on AHI index, but some more subtle disease indices. An ongoing and unresolved argument is pending, what are predictors of EDS, main culprits being sleep fragmentation due to arousals and intermittent hypoxemia, but also metabolic comorbidities, e.g. glucose intolerance^10,11^. Moreover, what is the contribution of EDS to prevalent cardiovascular or metabolic complications of OSA and why effects of CPAP treatment throughout the spectrum of the disease are so inconsistent^12^.

Based on clinical observation, we noted that some patients with substantial daily sleepiness presented with relatively low AHI calculated for total sleep, while SDB was confined to REM sleep (REM-dependent OSA, REM-OSA). Additionally, it has been shown that REM-dependent OSA is associated with cardiovascular and metabolic complications. Mokhlesi *et al*. reported that incidence of hypertension was strongly related to SDB in REM sleep^13^. Similarly, Grimaldi *et al*. found that worsening of glycemic control in type 2 diabetic patients was associated with SDB in REM sleep^14^. Thus, we hypothesise, that daily-sleepiness may be more related to SDB confined to REM sleep rather than occurring independently of sleep stage and the severity of the disorder defined by AHI alone. REM-OSA patients may have high AHI calculated exclusively for REM sleep, but due to usually fixed percentage of REM sleep in 20–25% range, their basal AHI calculated for total sleep time usually corresponds to mild or moderated disease. Therefore, to have a similar group for comparison in regard to AHI, but also other clinical metrics (e.g. age, sex, BMI) we decided to use mild and moderate (30 > AHI ≥ 5) sleep stage-independent OSA (nREM-OSA) as a control group. These patients are usually non-obese, presenting with positional disease, i.e. preponderance of SDB in the supine sleeping position.

Therefore, the aim of this study was to compare REM-OSA to nREM-OSA patients in respect to daily somnolence assessed by ESS^15^. We hypothesised, that SDB confined to REM results in greater EDS when compared to SDB not related to any sleep stage in patients with mild to moderate OSA severity.

## Materials and Methods

### Patients

The study group consisted of 1863 consecutive patients, who were referred to Sleep and Respiratory Disorders Centre with presumptive diagnosis of OSA between January 2011 and December 2016.

All patients included in the study were assessed and investigated by authors and underwent diagnostic polysomnography (PSG); while scoring PSG studies, the authors were blinded for the clinical data. Overall, the prevalence of OSA in our cohort as defined by AHI ≥ 5/h was 76.6%, n = 1427. Furthermore, patients were excluded from the study if their total sleep time was shorter than three hours, if sleep time either in lateral or supine position was shorter than half an hour or if total REM sleep was shorter than half an hour. At the initial visit before PSG, patients underwent a standard examination and ESS was performed. Patients, who were not able to answer all 8 questions from ESS were excluded from study. Furthermore, as REM-OSA patients usually suffer from mild to moderate disease individuals with severe form of nREM-OSA (AHI ≥ 30, usually non-position dependent OSA) were also excluded.

Next, based on PSG results, patients were assigned into two subgroups if they presented with one of two distinct OSA phenotypes: REM-OSA (defined by AHI_REM_/AHI_nREM_ ≥ 2 and AHI_REM_ ≥ 5/h) and nREM-OSA (defined by AHI_REM_/AHI_nREM_ < 1.5, and 30 > AHI ≥ 5/h) (Table 1). The latter group, usually, consisted of patients with positional disease, i.e. AHI_back_/AHI_side_ ≥ 2 (Table 1). We used AHI_REM_/AHI_nREM_ < 1.5 as an inclusion criterion for nREM-OSA to better differentiate it from REM-OSA with AHI_REM_/AHI_nREM_ ≥ 2, as this ratio is a continuum in the cohort and we wanted to have a good discrimination between study groups in this respect. Patients, who did not fulfil these criteria were excluded from the study. Finally, based on the aforementioned inclusion and exclusion criteria we found 102 patients with REM-OSA and 190 patients with nREM-OSA eligible for the study. More information on the process of patients’ selection can be found in the Supplementary Information section.

**Table 1:** Characteristics of patients, polysomnography related variables and ESS scores for REM-dependent and REM-independent OSA patients.

	REM-dependent OSA (n = 102)	REM-independent OSA (n = 190)	p-value
M:F ratio	42:60	96:94	0.091
Age at diagnosis	53.0 ± 10.8	54.1 ± 10.5	0.403
BMI [kg/m^2^]	32.0 ± 6.3	29.9 ± 4.5	**0.003**
TST [hours]	5.9 ± 1.0	5.8 ± 1.1	0.549
REM sleep stage time of TST [%]	21.7 ± 7.0	20.8 ± 6.7	0.274
Sleep time in supine position of TST [%]	55.4 ± 25.2	39.2 ± 19.8	**<0.001**
AHI_TST_	6.1 (3.4–10.0)	12.2 (7.6–18.8)	**<0.001**
AHI_back_/AHI_side_	2.0 (0.0–5.0)	17.9 (9.2–35.1)	**<0.001**
AHI_REM_/AHI_nREM_	6.0 (4.0–10.4)	0.6 (0.1–1.5)	**<0.001**
AHI_REM-related_	3.8 (1.9–5.7)	1.2 (0.4–2.7)	**<0.001**
AHI_nREM_	31.7 (19.0–53.2)	9.0 (4.8–15.0)	**<0.001**
AHI_back_	1.0 (0.3–3.0)	1.1 (0.0–5.2)	0.176
AHI_side_	5.7 (2.3–16.1)	16.0 (10.2–27.3)	**<0.001**
Arousal Index	13.5 (9.1–19.0)	19.0 (13.5–27.9)	**<0.001**
Mean desaturation SpO_2_ [%]	88.8(87.0–90.2)	88.3 (87.0–89.3)	0.345
Minimal SpO_2_ saturation [%]	82.1 (75.3–85.0)	81.4 (78.0–85.0)	0.531
ESS score	9.0 (6.0–11.0)	8.0 (4.8–11.0)	0.109
ESS score ≥ 10	31.4%	28.9%	0.689

BMI -body mass index; M:F – men to female ratio; AHI – apnea-hypopnea index; AHI_TST_ – AHI calculated for total sleep time; AHI_back_/AHI_side_ – the ratio of AHI calculated for sleep in the supine position to AHI in the lateral position; AHI_REM_/AHI_nREM_ - the ratio of AHI calculated for REM sleep and non-REM sleep; AHI_REM-related_ – all apneas and hypopneas that occurred in REM sleep divided by total sleep time; all data are presented as a median, lower and upper quartile; ESS – Epworth sleepiness scale; SpO_2_ – % of haemoglobin O_2_ saturation; TST – total sleeping time.

### Polysomnography

Patients were admitted to the sleep lab at 21:00 hours (±0.5 hour) and underwent physical examination (measurement of body mass, height, heart rate and blood pressure). A standard nocturnal polysomnography was performed by recording the following channels: electroencephalography (C4\A1, C3\A2), chin muscles and anterior tibialis electromyography, electrooculography, measurements of oro-nasal air flow (a thermistor gauge), snoring, body position, respiratory movements of chest and abdomen (piezoelectric gauges), unipolar electrocardiogram and haemoglobin oxygen saturation (SaO_2_) (Sleep Lab, Jaeger – Viasys, Hoechberg, Germany). Sleep stages were scored according to the criteria based on 30 s epoch standard^10,11^.

Apnea was attained with the reduction of air flow to less than 10% of the baseline for at least 10 s. Hypopnea was defined as at least 30% reduction of air flow for at least 10 s, accompanied by over 3% decrease in SaO_2_ or an arousal. EEG arousals were scored according to AASM guidelines^16^.

The study was conducted in accordance with the amended Declaration of Helsinki. All patients gave their informed consent to the sleep study. Ethics Committee of the Medical University of Lodz approved the study protocol (RNN/23/15/KE).

### Statistical analysis

The data were analysed with Statistica 12 software. Data distribution was tested with the Shapiro-Wilk test. The Student’s T-test or Mann Whitney U were used to compare continuous variables in case of normal and non-normal distribution of data, respectively. The frequencies were compared with Chi^2^ test. Subsequent post-hoc ANCOVA analysis was performed including covariates, which were different between study groups. Results are reported as a mean ± standard deviation for normally distributed data and as a median with interquartile range (LQ – UQ) for non-normally distributed data. P < 0.05 was considered statistically significant.

## Results

Out of 1863 patients, 292 were included in the final analysis of which 137 (46.9%) were men and the mean age of the group was 53.7 ± 10.6 years. Hundred and two individuals fulfilled criteria for REM-OSA and 190 for nREM-OSA.

There were no differences between the groups regarding sex, age, total sleep time (TST) or percentage of REM sleep. Groups differed regarding BMI and time spent in supine position during sleep. Characteristics of patients with REM-OSA and nREM-OSA is shown in Table 1.

AHI was 2 times higher for nREM-OSA than REM-OSA group (p < 0.001). To measure the impact of SDB occurring in REM sleep we calculated REM related AHI (AHI_REM-related_) dividing the number of REM apneas/hypopneas by TST and it was over 3 times higher in REM-OSA (p < 0.001). The polysomnography related variables and daytime sleepiness for REM- and nREM-OSA groups are presented in Table 1.

We subsequently divided the study groups into two subgroups: ESS < 10 and ESS ≥ 10 to find any factors that may be related to daily sleepiness. We observed no difference in the percentage of individuals scoring in the scale 10 or more between the groups. Moreover, metrics related to OSA, i.e. number of apneas/hypopneas, arousal index and desaturation parameters did not differ within subgroups (low vs high ESS) despite different median ESS scores. The differences in AHI and arousal index were similar between corresponding subgroups (low ESS REM-OSA vs nREM-OSA and high ESS REM-OSA vs nREM-OSA) to the differences noted between whole groups (Table 1). The results of this analysis are presented in Table 2.

**Table 2:** Comparison of study subgroups with or without EDS defined by ESS ≥ 10 within and between REM-OSA and nREM-OSA phenotypes.

	REM-dependent OSA (n = 102)		REM-independent OSA (n = 190)		p			
	ESS < 10 (1)	ESS ≥ 10 (2)	ESS < 10 (3)	ESS ≥ 10 (4)	1 vs 2	3 vs 4	1 vs 3	2 vs 4
N (%)	61 (59.8)	41 (40.2)	122 (64.2)	68 (35.8)	NA	NA	0.227	0.227
M:F	21/40	21/20	61/61	35/33	0.147	0.846	**0.046**	0.786
Age at diagnosis	52.38 ± 12.0	54.0 ± 8.8	54.4 ± 11.3	53.7 ± 9.0	0.460	0.685	0.276	0.868
BMI [kg/m^2^]	30.9 ± 5.5	33.7 ± 7.1	29.9 ± 4.2	30.0 ± 5.1	**0.027**	0.902	0.198	0.004
TST [hours]	5.9 ± 1.1	5.9 ± 5.9	5.8 ± 1.0	5.7 ± 1.2	0.750	0.888	0.483	0.737
REM sleep stage time of TST [%]	22.3 ± 7.7	21.0 ± 5.7	20.1 ± 6.5	22.2 ± 6.8	0.369	**0.034**	**0.045**	0.340
AHI_TST_	5.9 (3.3–10.7)	6.2 (3.4–9.1)	12.3 (7.6–19.0)	10.0 (7.6–18.0)	0.940	0.501	**<0.001**	**<0.001**
AHI_REM_/AHI_nREM_	5.5 (3.5–11.1)	6.0 (4.1–8.4)	0.64 (0.12–1.72)	0.45 (0.2–1.2)	0.542	0.580	**<0.001**	**<0.001**
AHI_REM_–_related_	15.9 (10.2–24.4)	18.9 (10.4–28.9)	6.3 (2.2–19.2)	4.4 (2.2–11.1)	0.921	0.221	**<0.001**	**<0.001**
AHI_nREM_	2.7 (1.3–6.0)	2.6 (1.2–4.5)	12.6 (7.5–18.8)	12.1 (8.0–17.1)	0.616	0.642	**<0.001**	**<0.001**
AHI_back_	10.0 (4.7–16.5)	9.0 (4.7–14.0)	32.0 (20.2–54.1)	30.7 (16.1–52.2)	0.790	0.712	**<0.001**	**<0.001**
AHI_side_	1.5 (1.0–6.0)	1.0 (0.0–4.0)	1.0 (0.5–3.0)	1.0 (0.2–2.2)	0.375	0.679	0.110	0.809
Arousal index	12.4 (9.0–20.8)	14.1 (10.9–17.3)	19.1 (14.5–28.6)	19.0 (12.8–25.0)	0.542	0.245	**<0.001**	**0.003**
Mean desaturation SpO_2_ [%]	88.8 (87.0–90.4)	81.5 (72.9–84.0)	81.0 (77.0–85.0)	88.7 (87.0–90.0)	0.191	0.464	0.220	0.449
Minimal SpO_2_ saturation [%]	83.0 (76.5–85.8)	81.5 (72.9–84.0)	81.0 (77.0–85.0)	82.0 (79.9–84.7)	0.238	0.756	0.436	0.511
ESS score	7.0 (5.0–8.0)	12.0 (11.0–14.0)	5.0 (4.0–7.0)	13.0 (11.0–14.75)	**<0.001**	**<0.001**	**0.004**	0.281

The table presents means ± SD, or medians (interquartile range) depending on distribution; NA – not applicable.

Subsequent ANCOVA analysis (shown in Table 3) included variables that were different between groups: BMI, percent of TST spent in REM stage and percent of TST spend in supine position and were chosen as covariates for the purpose of the analysis. The analysis showed no difference between groups regarding EDS (p = 0.308), while severity of the disorder (AHI) remained statistically different (p < 0.001).

**Table 3:** ANCOVA analysis adjusted for confounding factors.

		AHI	Epworth
p-value		**<0.001**	0.063
Least squared means	REM dependent	7.267	8.656
	REM independent	14.236	8.137
F-value		13.928	2.258

*Confounding factors: BMI (body-mass index), percent of total sleep time spent in REM (rapid eye movement) sleep stage, percent of total sleep time spent in the supine position.

AHI – apnea-hypopnea index.

## Discussion

We found that despite twice higher AHI in nREM- vs REM-OSA, both groups presented with similar daytime sleepiness based on ESS score, after adjusting for confounding variables. This result seems relevant as daily sleepiness represents an important aspect of OSA morbidity. This is not explicitly the confirmation of our hypothesis, because the study groups revealed similar daily sleepiness at different AHI levels, while we suspected the converse, i.e. the similar AHI levels but different ESS scores.

In previously published papers on relation of OSA severity and EDS, majority of them reported no correlation between the AHI index and ESS score^9,17^; similarly, we found no such correlation (data not shown). Therefore, other factors resulting in daytime sleepiness in OSA patients need to be addressed. As our study demonstrated, the ESS as a measure of day-time sleepiness was similar in study groups, despite lower AHI in REM-OSA group. Contrary to our findings, Punjabi *et al*.^18^ reported an increased risk of high EDS associated with SBD in non-REM sleep. However, EDS in the study was assessed by the Multiple sleep latency test, which even though considered to be more objective, focuses on different aspect of daily sleepiness than ESS. Furthermore, it has been suggested that EDS rather than being the effect of REM-dependent OSA, might be a result of lower O_2_ saturation during sleep among these patients^19^. However, we have not found any differences between our study groups regarding O_2_ saturation parameters, e.g. the mean SpO_2_ of desaturations and minimal SpO_2_, (Table 1, other saturation parameters not shown), which was also the case in other studies^20^. Similarly, when a subgroups with low and high EDS were compared, the difference in EDS could not be explained by saturation parameters or arousal index, as they did not differ (Table 2).

Thus, some other hypotheses are to be considered. It is plausible that non-interrupted REM is essential to the restorative properties of sleep; SDB in REM leads to arousals and as a consequence REM sleep fragmentation. The increased EDS in REM-OSA may be due to disruption of this phase continuity by SDB related arousals. Arousal index calculated for the total sleep time was lower in REM- vs nREM-OSA group, therefore, it could not be responsible for daily somnolence. Still, when we calculated AHI using only apneas/hypopneas that occurred in REM sleep, it was 3-fold higher in REM-OSA than in nREM-OSA, which could explain similar symptomatology despite lower total AHI. Hence, this indirectly supports the notion the integrity of REM sleep may be essential for refreshing properties of sleep and apneas/hypopneas occurring in this sleep stage may have greater impact on EDS.

The study groups were well matched regarding the clinical variables (Table 1) with the exception of BMI. REM-OSA presented with the mean BMI 2 kg/m^2^ higher than nREM-OSA group. It seems rather infeasible that this small difference might have accounted for the difference in daily-sleepiness, as ANCOVA analysis excluded this confounding effect.

Our study showed roughly 5.5% of patients suffering from OSA presented a pure REM-dependent phenotype, while, some studies suggested that the prevalence of this phenotype might be higher, reaching up to 10%^21^. This lower prevalence of REM-OSA phenotype might be related to strict inclusion criteria for this group that we used. There is evidence to support OSA patients with higher EDS respond better to CPAP treatment, reducing other comorbidities (e.g. high blood pressure)^22^ and improving quality of life^21,23^. Therefore, due to a higher morbidity in REM dependent OSA it may be justified to consider CPAP treatment at lower AHI levels.

### Limitations of the study

In order to compare REM-OSA and nREM-OSA phenotypes we enrolled patients with SDB confined to REM sleep (AHI_REM_/AHI_nREM_ ≥ 2) and a control group with SDB not dependent on sleep stage (AHI_REM_/AHI_nREM_ < 1.5) within similar, mild to moderate, AHI range. Nevertheless, it should be underlined that the group selection was not perfect due to intrinsic properties of nREM-OSA phenotype. While, pure REM-OSA does exist, with patients presenting with SDB exclusively in REM sleep, in nREM-OSA SDB occurs in REM and nREM sleep to the similar extent, thus there is no a pure nREM-OSA phenotype to use for comparison. Therefore, we compared REM-OSA phenotype with a mixed one, i.e. no sleep stage dependent one. However, the simulated AHI calculated exclusively from all apneas/hypopneas that occurred only in REM sleep was 3-times higher in REM-OSA than in nREM-OSA which defends our group selection criteria and study design.

Matching the groups with respect to AHI and other clinical variables was another problem affecting the study design and analysis. REM-OSA patients usually have similar night to night AHI which depends mainly on rather constant REM sleep percentage and is in the mild-moderate range. In mild to moderate nREM-OSA group it can be highly variable and depends on percentage of sleep spent in a supine position, as majority of these patients have positional OSA with the ratio AHI_back_/AHI_side_ ≥ 2. This implicates that their disease is better described by AHI range (from low AHI_side_ to high AHI_back_) than a single value and night to night AHI in this group is variable and it cannot be predicted from a single PSG night. Therefore, our groups differed in respect to AHI but had similar daily sleepiness per ESS, which was converse to the study hypothesis.

In conclusion, this study contributes to the pending discussion on predictors of OSA morbidity. Apart from implicated quantitative indices of disease severity, e.g. AHI, arousals or desaturations, we posit that qualitative differences in OSA phenotypes related to SDB occurring in different sleep stages may independently explain the severity of daily sleepiness.

## Supplementary information

Supplementary Information
