# Supplementary material for: Association between excessive daytime sleepiness, REM phenotype and severity of obstructive sleep apnea
Source: Sci Rep. 2020 Jan 8;10:34. doi: 10.1038/s41598-019-56478-9 (PMC6949253; doi:10.1038/s41598-019-56478-9)
Supplement: Supplementary file 1 — Supplementary Information [file 41598_2019_56478_MOESM1_ESM.docx]

Title: Association between excessive daytime sleepiness, REM phenotype and severity of obstructive sleep apnea

Authors: Agata Gabryelska, Piotr Białasiewicz

Department of Sleep Medicine and Metabolic Disorders

Medical University of Lodz

Mazowiecka 6/8, 92-215 Lodz

Corresponding Author

Agata Gabryelska

Department of Sleep Medicine and Metabolic Disorders

Medical University of Lodz

Mazowiecka 6/8, 92-215 Lodz, Poland

Email: agata.gabryelska@gmail.com

Tel: +48 660 796 004

Supplementary information on the inclusion and exclusion criteria applied for the study groups

It is of paramount importance to define REM- and nREM-OSA groups for the purpose of the study. AHI_REM_/AHI_nREM_ ratio, as expected, was a continuum in our cohort. Therefore, we decided to lower the ratio of AHI_REM_/AHI_nREM_ to less than 1.5 from traditionally, but still arbitrary used 2.0 for nREM-OSA group, to have well separated, unmixed groups in respect to this ratio.

The prevalence of OSA in our cohort was 76.6% (1427 patients) as defined by AHI of at least 5. To explain why we didn’t just divided the whole cohort into REM and NREM OSA based on traditionally used cut-off point of 2 for REM / NREM AHI ratio, and why so many patients were found not eligible for the study, please find below the graphic depiction of 5 major OSAS phenotypes:

Fig. 1 - Graphic presentation of OSA phenotypes

**A**

sleep stage

body position

apnea/hypopnea

NREM

NREM

REM

REM

supine

lateral

A - Non-position and non-sleep stage dependent OSA with both ratios: supine / lateral and REM / NREM AHI less than 2.0 (actually less than 1.5). These patients were excluded from the study, because they usually suffered from severe OSA, and their AHI would be at least few times higher than that found in REM-OSA.

B - Position and non-sleep stage dependent OSA with supine / lateral AHI ratio greater than 2.0 and REM/NREM AHI less than 1.5. These patients were included in the study as a control for REM-OSA, because they usually had mild to moderate disease. In this group AHI changes night to night depending on the percentage of time they spend sleeping in the lateral or supine position. Overall, their AHI is in similar range to REM-OSA, but nevertheless proved to be twice that high in our selected cohort.

**B**

sleep stage

body position

apnea/hypopnea

NREM

NREM

REM

REM

supine

lateral

C - Pure REM dependent OSA with supine / lateral AHI ratio less than 1.5 and REM / NREM AHI greater than 2.0 These patients were included in the study as a part of REM- OSA group.

**C**

sleep stage

body position

apnea/hypopnea

NREM

NREM

REM

REM

supine

lateral

D - Mixed OSA phenotype; non-sleep stage dependent in the supine sleeping position and REM dependent in the lateral sleeping position. These patients were excluded from the study.

**D**

sleep stage

body position

apnea/hypopnea

NREM

NREM

REM

REM

supine

lateral

**E**

sleep stage

body position

apnea/hypopnea

NREM

NREM

REM

REM

supine

lateral

E - Position and REM dependent OSAS with supine / lateral AHI ratio greater than 2.0 (but only because of the occurrence of SDB in REM supine) and REM / NREM AHI ratio greater than 2.0. These patients were included in the study as a part of REM-OSA group.

Moreover there were some additional exclusion criteria:

If for instance, a patient slept only in the lateral position and presented with pure REM-OSA, he/she was excluded because actually might belonged to mixed phenotype (D).

If patient from group E slept only in the lateral position, he/she was excluded because one didn’t know that apneas were confined to REM in the supine position (a false negative). Therefor, we also excluded patients who slept less than 0.5 hour in either position or in REM sleep.

So, the process of patients selection was also based on the calculated ratio of REM / NREM AHI, but also on visual inspection of the individual PSG graphic summary. It is also a reason why so many patients despite OSA diagnosis were excluded from the study being not “pure enough” to be assign to one of the study groups.
